# Analyzing global utilization and missed opportunities in debt-for-nature swaps with generative AI

**DOI:** 10.3389/frai.2024.1167137

**Published:** 2024-02-05

**Authors:** Nataliya Tkachenko, Simon Frieder, Ryan-Rhys Griffiths, Christoph Nedopil

**Affiliations:** ^1^Smith School of Enterprise and the Environment, School of Geography and the Environment, University of Oxford, Oxford, United Kingdom; ^2^UK Centre for Greening Finance and Investment, University of Oxford, Oxford, United Kingdom; ^3^The Alan Turing Institute, Finance and Economics, London, United Kingdom; ^4^Department of Computer Science, University of Oxford, Oxford, United Kingdom; ^5^Department of Physics, University of Cambridge, Cambridge, United Kingdom; ^6^Griffith Asia Institute, Griffith University, Brisbane, QLD, Australia; ^7^Sim Kee Boon Institute for Financial Economics, Singapore Management University, Singapore, Singapore; ^8^Fanhai International School of Finance, Fudan University, Shanghai, China; ^9^Belt and Road Initiative Green Coalition, Ministry of Ecology and Environment of People's Republic of China, Beijing, China

**Keywords:** DNS, retrieval augmented generation, nature-based solutions, sustainable credit finance, nature finance, adaptation finance, generative AI, GPT-4

## Abstract

We deploy a prompt-augmented GPT-4 model to distill comprehensive datasets on the global application of debt-for-nature swaps (DNS), a pivotal financial tool for environmental conservation. Our analysis includes 195 nations and identifies 21 countries that have not yet used DNS before as prime candidates for DNS. A significant proportion demonstrates consistent commitments to conservation finance (0.86 accuracy as compared to historical swaps records). Conversely, 35 countries previously active in DNS before 2010 have since been identified as unsuitable. Notably, Argentina, grappling with soaring inflation and a substantial sovereign debt crisis, and Poland, which has achieved economic stability and gained access to alternative EU conservation funds, exemplify the shifting suitability landscape. The study's outcomes illuminate the fragility of DNS as a conservation strategy amid economic and political volatility.

## 1 Introduction and related work

Current global-scale nature financing instruments play a critical role in mobilizing resources for environmental conservation and climate change adaptation. Nature financing instruments are designed to address the funding gap in biodiversity and climate initiatives, estimated to be between 878.9 to 891.3 billion USD (EEA, 2022). For example, nature-based solutions (NbS) are increasingly funded through private-led financing initiatives and market-based mechanisms, such as carbon credits. Another increasingly relevant instrument focusing on private-led and public-led nature financing are debt-for-nature swaps (DNS). Recent trends in the aftermath of the emerging market debt crisis since 2019 indicate an increase in the volume and frequency of the application of DNS, underscoring their growing importance in global environmental finance (UNDP, 2022). The evolution of DNS from smaller, grant-like arrangements covering bilateral sovereign debt to large-scale, impactful financial instruments covering sovereign bonds marks a significant shift in conservation financing, offering a viable solution to simultaneously address environmental conservation and sovereign debt sustainability. The reach of these swaps is also expanding, with countries like Ecuador and Sri Lanka exploring large-scale DNS type sovereign debt refinancing. Ecuador, for example, is in talks for an 800 million USD refinancing to support conservation efforts (Duarte and White, 2023), and Sri Lanka is discussing a one billion USD swap (Costa, 2023). These initiatives highlight how DNS are increasingly being considered in countries with significant biodiversity but facing fiscal distress.

DNS have evolved significantly since their inception in the late 1980s (Sheikh, 2016). Initially resembling grants, modern DNS involve sovereign bond refinancing linked to conservation performance indicators where high-risk and high-interest bonds and loans are re-financed with newly issued and lower interest bonds supported by a mix of private and development finance institutions' investments or guarantees (Hrynik, 1990; Patterson, 1990). This enables private creditors to participate in debt restructuring with a nature conservation aspect. These transactions have focused on conservation financing for ecosystems like coral reefs and biodiverse coastlines, with significant deals taking place in various parts of the world. One notable example is the 2021 debt-for-nature swap in Belize (Owen, 2022), a major step up from the earlier 21.6 million USD Seychelles deal in 2018 by The Nature Conservancy (TNC) (Booth and Brooks, 2023). Belize's swap directed financing toward the world's second-largest coral reef, reducing its debt by over 12% of GDP. The deal involved a TNC subsidiary lending funds to Belize to buy back a 553 million USD “superbond” at a discounted rate, financed by a 364 million USD blue bond issuance. This deal significantly improved Belize's credit rating and demonstrated the potential of DNS to achieve both environmental and fiscal benefits.

Earlier studies identified that DNS as a financial instrument have a strong potential when compared to other levers. Specifically, they can create a long-term funding commitment for conservation projects, which can be more sustainable than one-off grants or donations (Post, 1990; Sadler, 1990; Asiedu-Akrofi, 1991; Dillon, 1991; Greener, 1991; Rosebrock and Sondhof, 1991; Sarkar and Ebbs, 1992; Sher, 1993; Thapa, 2000). These swaps can turn a liability (i.e., debts) into an asset (i.e., conserved natural resources), thus leveraging existing financial obligations for positive impact, and often come with the backing of governments, ensuring a level of political commitment that other private financing mechanisms may lack. The predetermined nature of the financial commitments ensures consistent funding, unlike donor contributions, which can be volatile and unsustainable. However, this model is largely applicable to debtor countries with rich biodiversity, limiting its global applicability (Fuller, 1989; Lewis, 1999). Nonetheless, debt-for-nature agreements can be complex to negotiate as they often involve multiple stakeholders, including governments, financial institutions, and NGOs. And while impactful when success is achieved, the scale of funds for biodiversity protection generated through DNS can be relatively modest as compared to other instruments like carbon trading and payments for ecosystem services (PES) (Lachman, 1988; Bedarff et al., 1989; Model, 1990; Moltke and DeLong, 1990; Potier, 1991; Klinger, 1994; Wee, 1994; Chambers et al., 1996; Macekura, 2016; Nedopil et al., 2024).

Hence, despite their potential, DNS have not been widely adopted or scaled up as envisioned (Duarte and White, 2023). The mentioned complexity of DNS stems from various factors. One of the primary challenges identified is the high transaction costs incurred due to the involvement of multiple stakeholders, including debtors, creditors, donors, and NGOs. These stakeholders often have different objectives and interests, leading to prolonged preparation and negotiation processes that can reduce the efficiency of DNS implementation. Conflicts can also arise when environmental protection measures infringe on the interests of local or indigenous communities, potentially affecting the ability of DNS mechanisms to promote national and local wellbeing (Patterson, 1990; Greener, 1991).

Several sources (Hansen, 1988; Gockel and Gray, 2011; Hassoun, 2012) point to the reluctance or inability of creditors to offer such swaps and debtor countries' unwillingness to accept them, often due to concerns about sovereignty or negative social and economic impacts. The complex and lengthy negotiation processes involved, requiring agreement among diverse stakeholders with varying interests, represent another significant challenge. Furthermore, the swaps typically involve only a fraction of a country's total external debt, limiting their impact on reducing debt burdens or improving creditworthiness. They also often fail to address the root causes of environmental degradation, such as weak governance or market failures, thus not ensuring long-term sustainability outcomes.

Despite the extensive knowledge accumulated on DNS, there remains a significant gap in comprehensively evaluating their success, particularly in addressing funding gaps that hinder continuous nature recovery at scale (Costa, 2023). To bridge this gap, a global summarization approach of vast data resources is crucial. For this task, we therefore use an emerging generative artificial intelligence (AI) technology, Large Language Models (LLMs), which are capable of efficient data synthesis from various sources. These AI models, capable of analyzing and synthesizing data at an unprecedented scale, are instrumental in uncovering trends and insights, particularly in identifying funding gaps and inconsistencies that could have disrupted the continuity of nature recovery efforts. By “injecting” (Martino et al., 2023) additional domain-specific information sources from various NGOs and development finance institutions into an LLM base model, we re-focus our analysis on pinpointing best practices and potential pitfalls while also uncovering missed opportunities in global nature debt finance.

## 2 Methodology

DNS represent a unique topical intersection of finance and environmental conservation, making them an ideal candidate for domain-specific cases of LLMs deployment, which can fully stress-test models' performance regarding accuracy and specificity. LLMs like GPT-4 (OpenAI, 2023) are trained on vast datasets sourced from the web, encompassing a wide range of topics, languages, and styles (Zhao et al., 2023). This extensive training enables them to generate human-like text and respond to a variety of queries with impressive versatility. However, a significant drawback is their tendency to “hallucinate” (Maynez et al., 2020; Ji et al., 2023), meaning they sometimes generate plausible but incorrect or nonsensical information. This issue is compounded by the fact that LLMs can inadvertently propagate misinformation present in their training data, necessitating careful monitoring and intervention to ensure the accuracy and reliability of their outputs.

**Figure 1:**
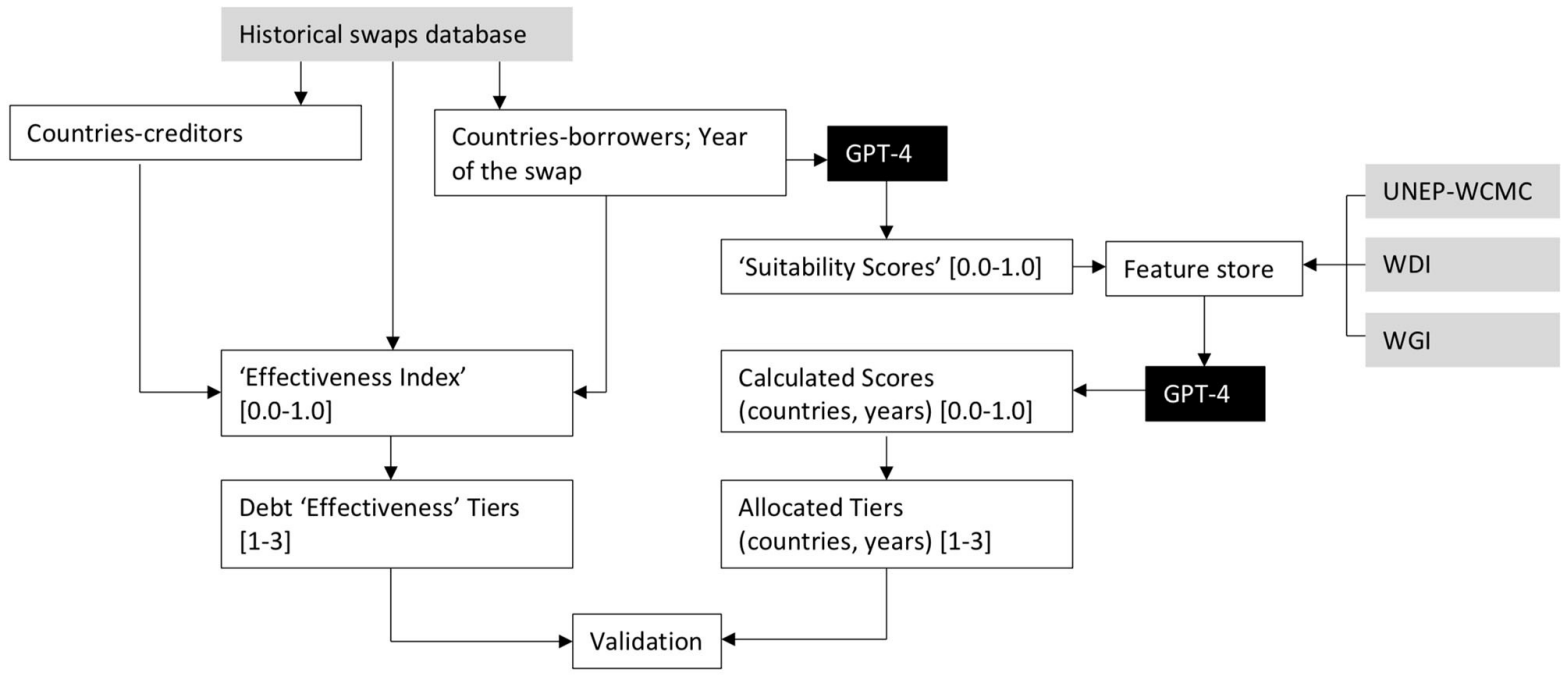
Flowchart, describing connectivity between input datasets with GPT-4 prompts, processed intermediate results, and the final validation output. The nodes in gray denote primary data sources and the nodes in black generative AI models.

**Figure 2:**
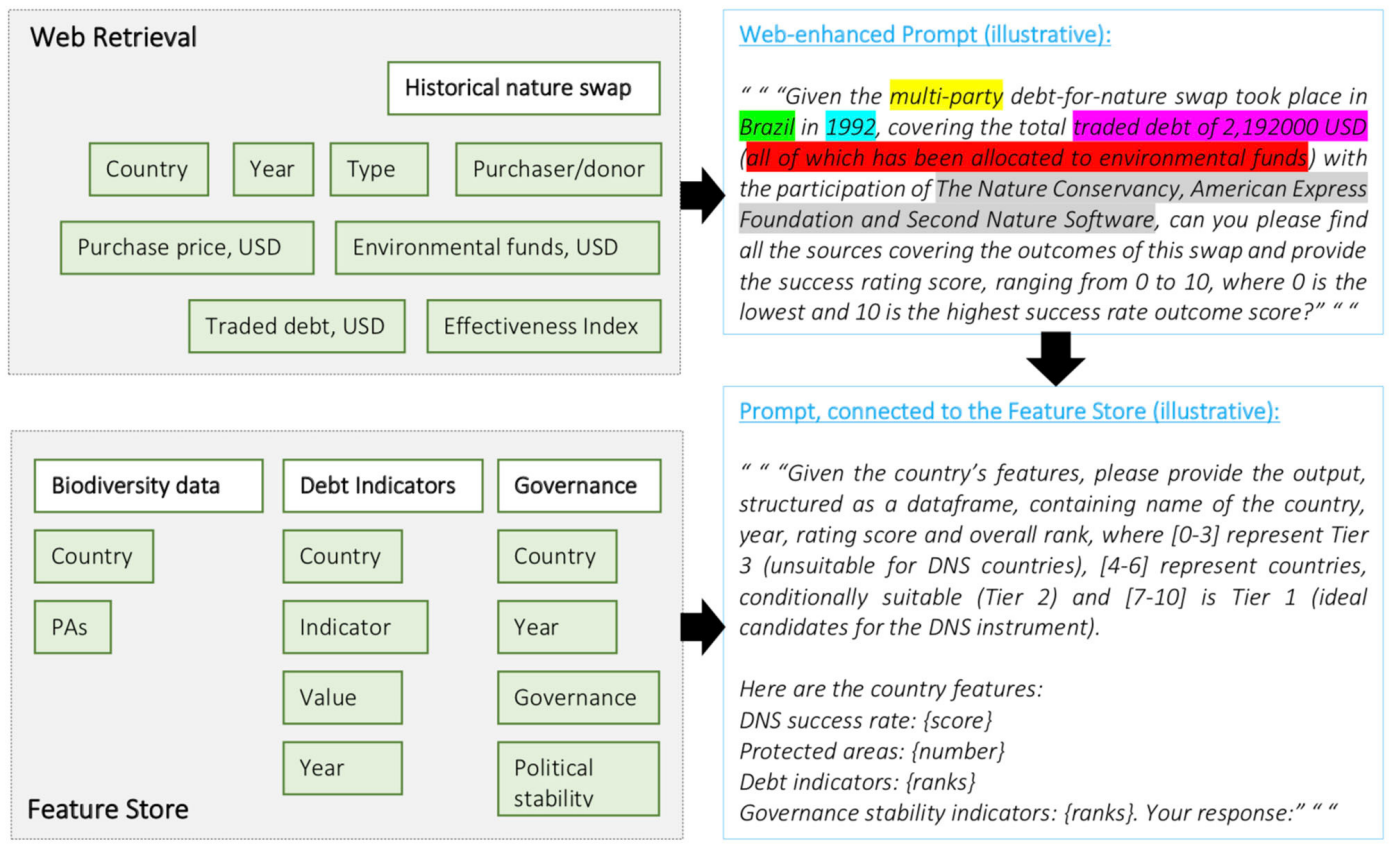
Illustrative examples of the sequential prompt-tuning inputs using our RAG approach.

In order to achieve acceptable results, several studies (Feldman et al., 2023; Martino et al., 2023; Ram et al., 2023; Rawte et al., 2023; Siriwardhana et al., 2023) already mentioned a few advantages of the prompt-tuning methods, such as retrieval augmented generations (RAGs), when dealing with inaccurate LLMs' outputs and mitigating downstream error propagation. It has been already acknowledged that without appropriate guardrails, hallucinatory LLMs in policy and financial services design can potentially lead to significant negative consequences, such as propagation erroneous results or misguided policy decisions. Properly aligned, LLMs can, however, offer valuable insights into the mechanics, benefits, and challenges of DNS, hence ensuring precision and accuracy remain paramount. Keeping in mind these constraints, we designed a specific adaptation of the RAG in the LangChain tool, which is capable of assembling various text sources into a single execution pipeline (see Figures 1, 2). To aid understanding, we outline here how parts of the methodology work in the case of the 1992 Brazil swap as an illustrative example, which had an estimated effectiveness index of 0.9 (which represents the proportion of the total budget actually allocated to environmental funds to the total value of traded debt in USD).

The methodology involves a two-step process using GPT-4 integrated with LangChain for document retrieval and processing. First, the authors provide a query, which GPT-4 uses to collect relevant documents from the web: “Debt-for-nature swap in Brazil, in 1992”. This collection is facilitated by LangChain, a tool that extends language models' capabilities by interfacing with external data sources. Once the documents are retrieved, we process the text to create embeddings, which are vector representations of the document's content. These embeddings capture the semantic and contextual nuances of the text, enabling a machine-readable understanding of the documents' content. The embeddings are then sent to a vector store, a specialized database designed for storing and querying high-dimensional vectors efficiently. This vector store allows for rapid retrieval and comparison of embeddings. To generate a success/suitability score, we call upon the vector store to retrieve relevant embeddings based on our query context. We then compute prompt-defined measures between the query embeddings and the document embeddings stored in the vector store. Instance success calculation is achieved by comparing the similarity measures to a predefined threshold. If the similarity measure is high, indicating a close match between the query and the documents' contexts, a score close to 1 is assigned, denoting high success. Conversely, a low similarity measure results in a score closer to 0, indicating a less successful match2. This entire process is automated. Classification part of the methodology employs GPT-4 to interface with the Feast feature store, a system used for managing and serving machine learning features. The feature store contains three merged data frames: one with the number of protected areas per country, another with development indicators, and the third with governance rankings, all sourced from authoritative databases such as the World Bank, IMF, and UNEP-WCMC.

Initially, GPT-4 processes a prompt to match scores and tiers, derived during the previous step, with the correct countries and years recorded in the feature store. It utilizes pre-established relationships within the data to ensure accuracy in the matching process. After successful matching, GPT-4 is tasked with extrapolating these features to complete the dataset for all countries and for the years ranging from 1980 to 2023. This involves predicting or estimating values for the years and countries with missing data. The generated data is then organized into tiers, which are systematically compared against a historical swaps database to validate the generated features. This validation step checks the coherence and accuracy of the newly created features with actual historical data.

### 2.1 Prompt engineering

Prompts are used to interact with LLMs to accomplish various semantic tasks, such as question answering, sentiment analysis, text summarisation, and many others. Prompt engineering for GPT-4 involves the design and formulation of prompts that effectively guide machine learning models to generate responses that are pertinent, insightful, and aligned with the specific requirements of a given task or inquiry. Unlike simpler models where the prompt might be a straightforward question or command, GPT-4's sophisticated architecture allows for a more nuanced interaction, enabling researchers to gain more precise and complex responses.

Emerging literature (Ekin, 2023; Lo, 2023; Wang et al., 2023; White et al., 2023) on prompt engineering and tuning lists several approaches:

- *Explicit contextualization*: provisioning of a detailed context before posing a question;
- *Question framing*: the way a question is framed can also affect the results. For example, open-ended questions may produce more exploratory answers, while closed-ended questions may yield concise, factual information;
- *Parameter tuning*: GPT-4 allows for adjustment of various parameters like temperature and max tokens. Lower temperature values (e.g., 0.2) make the output more focused and deterministic, while higher values (e.g., 0.8) make it more random and creative;
- *User-suggested contexts*: in advanced use cases, like the extraction of a DNS classification system, user-provided context can be crucial. This can include specific terminologies, datasets to consider, or even desired output formats;
- *In-context learning*: whilst GPT-4 itself is not fine-tunable, preparing a sequence of well-engineered prompts for a particular domain and testing them extensively can effectively “faux-train” the model to generate highly specialized responses;

The adopted prompt engineering in our analysis includes two sequential branches, which correspond to unstructured context assemblage from the web and its subsequent provisioning of the analog inputs into a prompt connected to the structured feature store (see Figure 1). To design our prompt we used two techniques from the list presented above, specifically explicit contextualization of historically recorded DNS cases (in order to request the decoder model to generate synthetic “Suitability Scores,” using rich semantic context extracted from the web), and user-suggested contextualization, which asks GPT-4 generator to extrapolate suggested scores toward the countries, which do not have existing records of DNS. In Figure 1, synthetically calculated scores and “Effectiveness Index” values [which represent real authoritative metrics, based on the proportion of the total traded debt (USD) to the environment-allocated funds (USD)] both are subsequently broken down into three equidistant tiers [1–3], to facilitate their final validation.

### 2.2 Retrieval augmented generator

A major shortfall of Large Language Models (LLMs) content generation remains “hallucination,” i.e., propagation of the false information in the output. This known deficiency is especially risky for enterprise use cases that require reliable, fact-based, controllable text generation at scale. To mitigate it, we utilize a technique called Knowledge Injection (KI) (Martino et al., 2023), where contextual data about the entities (DNS in our case) is collected from the authoritative web resources and then connected to a text-generation task via the prompt feature store.

#### 2.2.1 Web retrieval

Many LLM applications require user-specific data that is not part of the model's training set. In this process, external data is retrieved and then passed to the LLM when doing the generation step (see the relevant section from Figure 2).

The LangChain tool provides all the building blocks for RAG tasks, from simple to more complex. According to the documentation, it provides over 100 different document loaders (including webpages, pdf files, code) as well as integrations with the major API/data providers from all types of locations (private S3 buckets or public websites).

A key part of retrieval is fetching only the relevant parts of documents. This involves several transformation steps in order to best prepare the documents for retrieval. One of the primary ones here is splitting (or chunking) a large document into smaller chunks. Another key part of retrieval has become creating embeddings for documents. Embeddings capture the semantic meaning of the text, allowing us to quickly and efficiently find other pieces of text that are similar.

We used the records (African Natural Resources Management and Investment Centre, 2022) of historical swaps, to design the first part of our pipeline, which included prompt engineering for web retrieval using countries' names to extract all available information on the DNS agreements that have been covered by any of the authoritative sources (including academic papers, NGO reports, and authoritative environmental media outlets) (Ochiolini, 1990; O'Connor and Turnham, 1992; Lovei, 1995; Goldenman, 1998; OECD, 1998; Moye, 2002; OECD Task Force for the Implementation of the Environmental Action Programme for Central and Eastern Europe (OECD/EAP Task Force), 2002, 2004; Organisation for Economic Co-operation Development, 2002; U.S. Department of the Treasury, 2002a,b,c, 2003, 2004a,b,c, 2006, 2008, 2009, 2010, 2023; International Monetary Fund, 2003, 2014, 2016, 2022, 2023a,b,c; OECD Development Assistance Committee, 2003; Task Force for the Implementation of the Environmental Action Programme for Central and Eastern Europe (EAP Task Force) and Organisation for Economic Co-operation and Development (OECD), 2003a,b; Nigerian National Planning Commission, 2005; Organisation for Economic Co-operation and Development (OECD), 2005, 2006a,b,c, 2007, 2020; Paget et al., 2005; World Wildlife Fund, 2005, 2006; The Nature Conservancy, 2007, 2010, 2016, 2021; World Wide Fund for Nature (WWF), 2011, 2012, 2020; World Wildlife Fund (WWF), 2011a,b; IMF, 2013; Convention on Biological Diversity, 2017; World Bank, 2018a,b, 2019; Casado-Asensio et al., 2021; Deutz et al., 2021; Singh and Widge, 2021; The Nature Conservancy (TNC), 2021; Yue and Wang, 2021; Bala et al., 2022; Belianska et al., 2022; Georgieva, 2022; Owen, 2022; Adrian, 2023; Binnie, 2023; UK Parliament, 2023; United Nations Development Programme, 2023). To ensure credibility, we encoded the request to include brief summaries of the documents for easier verification. Extracted reports were subsequently converted to pdf files and fed into the vector database, containing domain-specific context for our chain prompt. The main function of this prompt was to analyze the entire corpus of the rich policy contexts and to return ranking values of the prospective suitability (the Python script of this pipeline will be made available upon request).

#### 2.2.2 Feature store

Feature stores are a concept from traditional machine learning that make sure data fed into models is relevant, and they help to ensure that user context is modular enough and easy to combine with the data contained within base LLMs.

Hence, the second part of the RAG included construction of the feature store with help of Feast, a customizable operational data system that re-uses existing infrastructure to manage and serve machine learning features to the real-time models. This service allowed us to structure our tabular inputs into a relational *.parquet* file-system, which could be seamlessly fed into the LangChain prompt template.

### 2.3 Data sources for retrieval-augmented generation

The literature points to the fact that usually, these swaps originate as a response to the need for conservation efforts in countries facing significant foreign debt burdens, with the recognition that many of these countries also harbor a large portion of the world's biodiversity. As a consequence, we decided to initiate a feature store containing three main datasets:

1. Concentration of protected areas: the first metric considered was the density and spread of protected areas in each country, using data from the International Union for Conservation of Nature - World Conservation Monitoring Center (IUCN-WCMC) (UNEP-WCMC and IUCN, 2023). Since the primary goal of DNS is to fund conservation activities, the effectiveness of these swaps usually depends on the viability of the conservation programs financed, the strength of the organizations and communities implementing these programs, and the long-term impact on conservation efforts.
2. Levels of external debt (the extent of the country's external financial obligations) data from the World Development Indicators, curated by the World Bank Data Store (The World Bank, 2023). The need for DNS often arises in countries with significant external debt, which may struggle to repay their debts fully. These situations lead to their debts being sold at a discount by commercial banks or governments and create an opportunity for conservation organizations to purchase the debt and use the proceeds for conservation activities.
3. Governance (the effectiveness and stability of the country's governing bodies), included in Worldwide Governance Indicators database (Kaufmann and Kraay, 2023) is another important feature as often the success of DNS hinges on the willingness and ability of the government of the indebted country to actively participate.

### 2.4 Defining countries' ranking

To assess the viability of countries for successful implementation of DNS, we decided to develop a relative ranking system (such as [0.0:1.0]), which would have enabled an easier comparison of the LLM-generated scores and the ground truth dataset. This approach was designed to capture a range of ecological, economic, and political factors, and to provide a comprehensive framework for evaluating each country's prospective suitability for the instrument. In this prompt, we avoided adding weights to each of the respective factors, mentioned above, and relied on the decoder's properties to imply the importance of each factor for individual countries using implicit stochastic mechanics of the generative process, taking place when context is fed into GPT-4 model.

**Figure 3:**
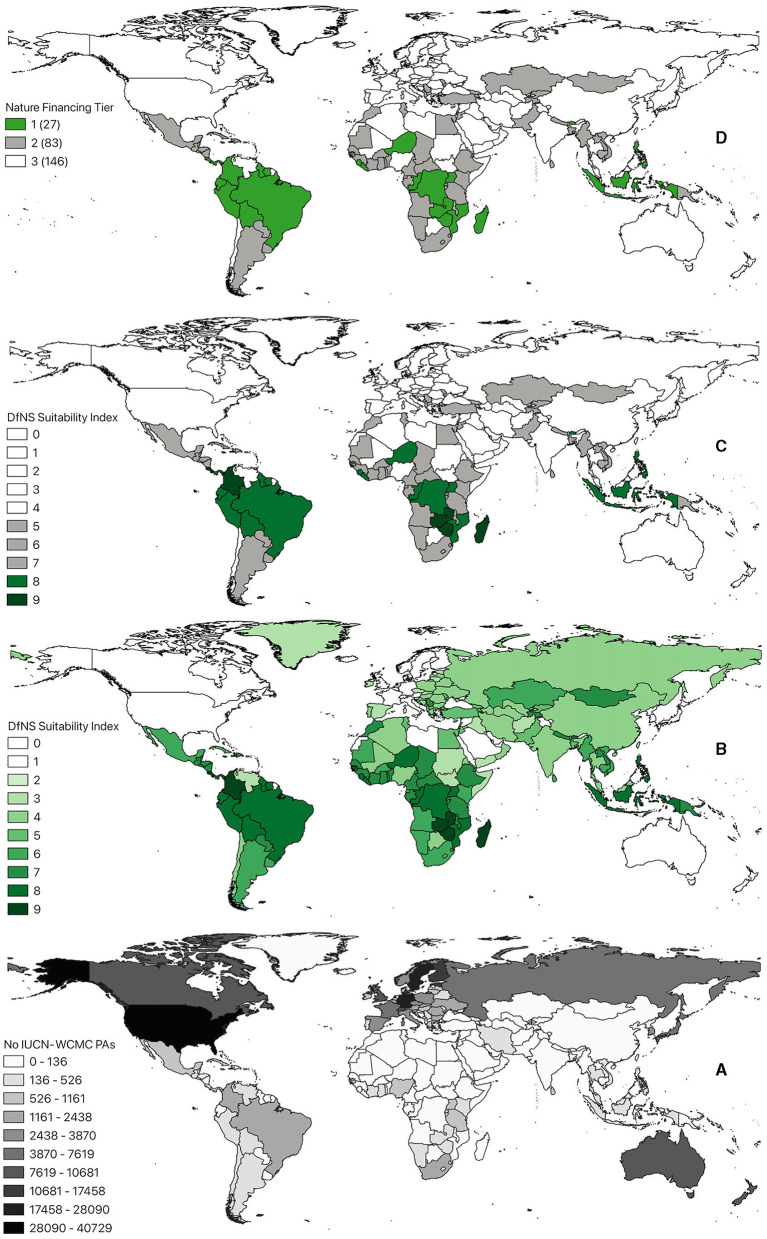
GPT-4 ranking of the countries, eligible for current DNS financial instruments: **(A)** Concentration of protected areas around the world (IUCN-WCMC); **(B)** Suitability of countries for DNS, assessed on a scale from 0 to 10 and normalized according to the levels of external debt, ecological significance, governance, and political stability of each country; **(C)** Derivation of three tiers of suitability for DNS, using 0–10 normalization scale; **(D)** Visualization of three tiers of countries, ranked according to their suitability for DNS, where 1 is the most suitable and 3 least suitable for this type of financing instrument tiers, respectively.

According to this system, countries are split into three equidistant tiers by the context-enriched GPT-4 prompt (see the relevant section from Figure 3):

1. Tier 1: highly suitable (scores 0.8–1.0)—These are countries with significant biodiversity, facing substantial external debt burdens, and a strong commitment to conservation efforts. They should have stable political environments, effective governance, and the capacity to manage conservation programs. These countries are typically able to leverage DNS effectively for both debt relief and significant environmental conservation.
2. Tier 2: conditionally suitable (scores 0.5–0.7)—These countries may have considerable biodiversity and external debt but face challenges like political instability, weaker governance, or limited capacity to manage conservation programs.
3. Tier 3: least suitable (scores 0.0–0.4)—Countries with low biodiversity significance, minimal external debt issues, unstable political environments, poor governance, and limited institutional capacity for managing conservation efforts are least suitable.

### 2.5 Validation

The validation process for the outputs of prompt-tuning in the context of GPT-4 or similar language models is crucial for ensuring the reliability and applicability of the model-generated information. We structured the validation process around quantitative and qualitative evaluation steps. This stage allowed for the identification of major discrepancies and refinement of prompt phrasing.

As part of the quantitative evaluation step, we utilized both accuracy metrics (precision, recall, and F1-score) against historical swaps database, maintained by the African Development Bank Group.

Human verification by the authors was performed to ensure that the outputs were accurate and contextually appropriate. They were evaluated against general alignment with the current understanding and data regarding DNS or any other subject matter, as reported by various authoritative and media sources. However, we note that it will be beneficial in the design of the future DNS with the help of AI to engage with experts in finance, conservation, and political science in order to complete human-in-the-loop assurance in the model's classifications and fair rankings of the candidate countries.

## 3 Results

**Figure 4:**
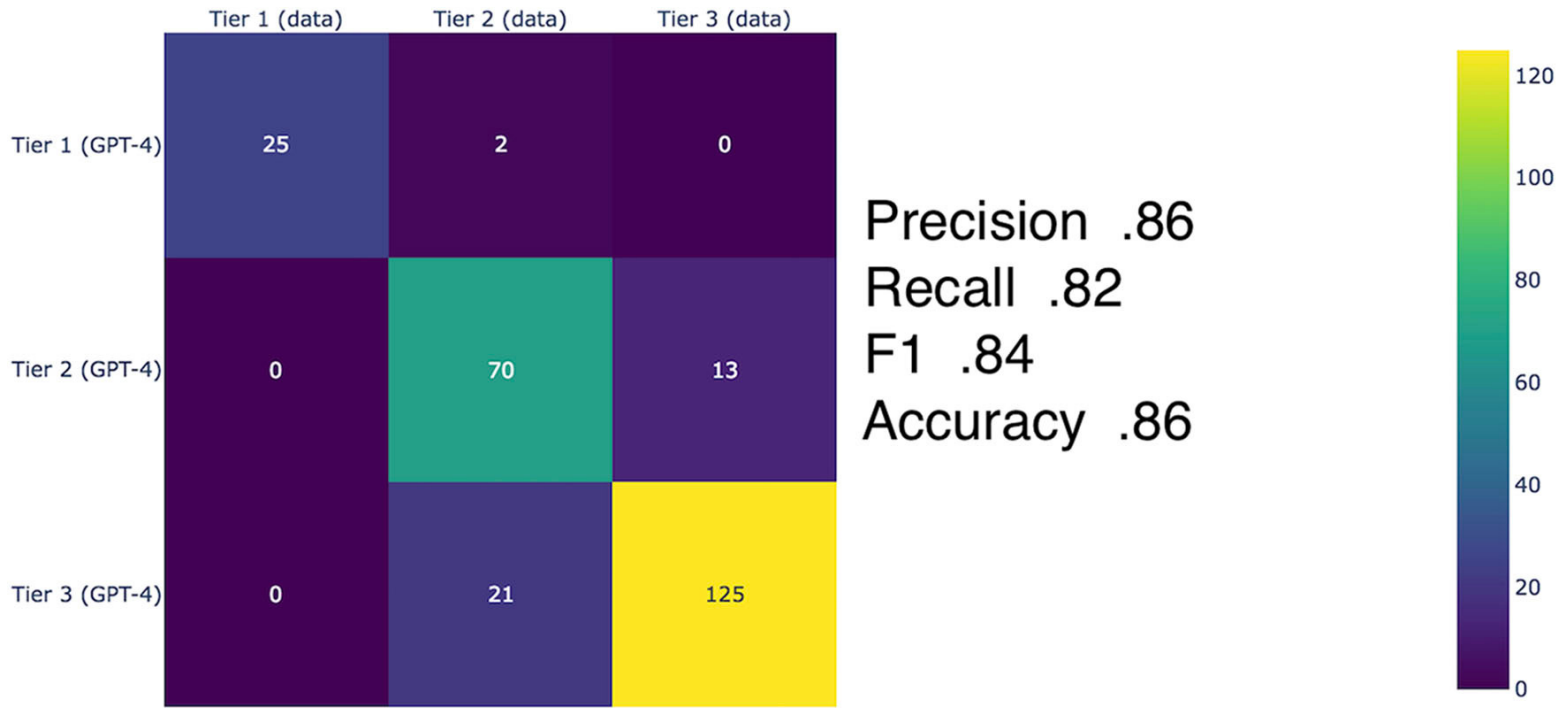
Matrix for RAG augmented GPT-4 scores (accuracy, precision, recall, and F1 Score) on the three country tiers. The aggregate accuracy score across all three tiers is 0.86 (0.93, 0.84, and 0.85 precision for Tiers 1, 2, and 3, respectively).

The overall validation of the results against historical swaps data demonstrated 0.86 accuracy (including precision 0.86, F1 score 0.84, and recall 0.82) (Figure 4). When broken down to the individual Tiers, precision scores of 0.93, 0.84, and 0.85 correspond to Tiers 1, 2 and 3, respectively. Although no significant observable difference is shown between Tiers 2 and 3, we may still be able to draw a conclusion that our model is well-suited for differentiating countries, which are best or least suited for DNS.

**Figure 5:**
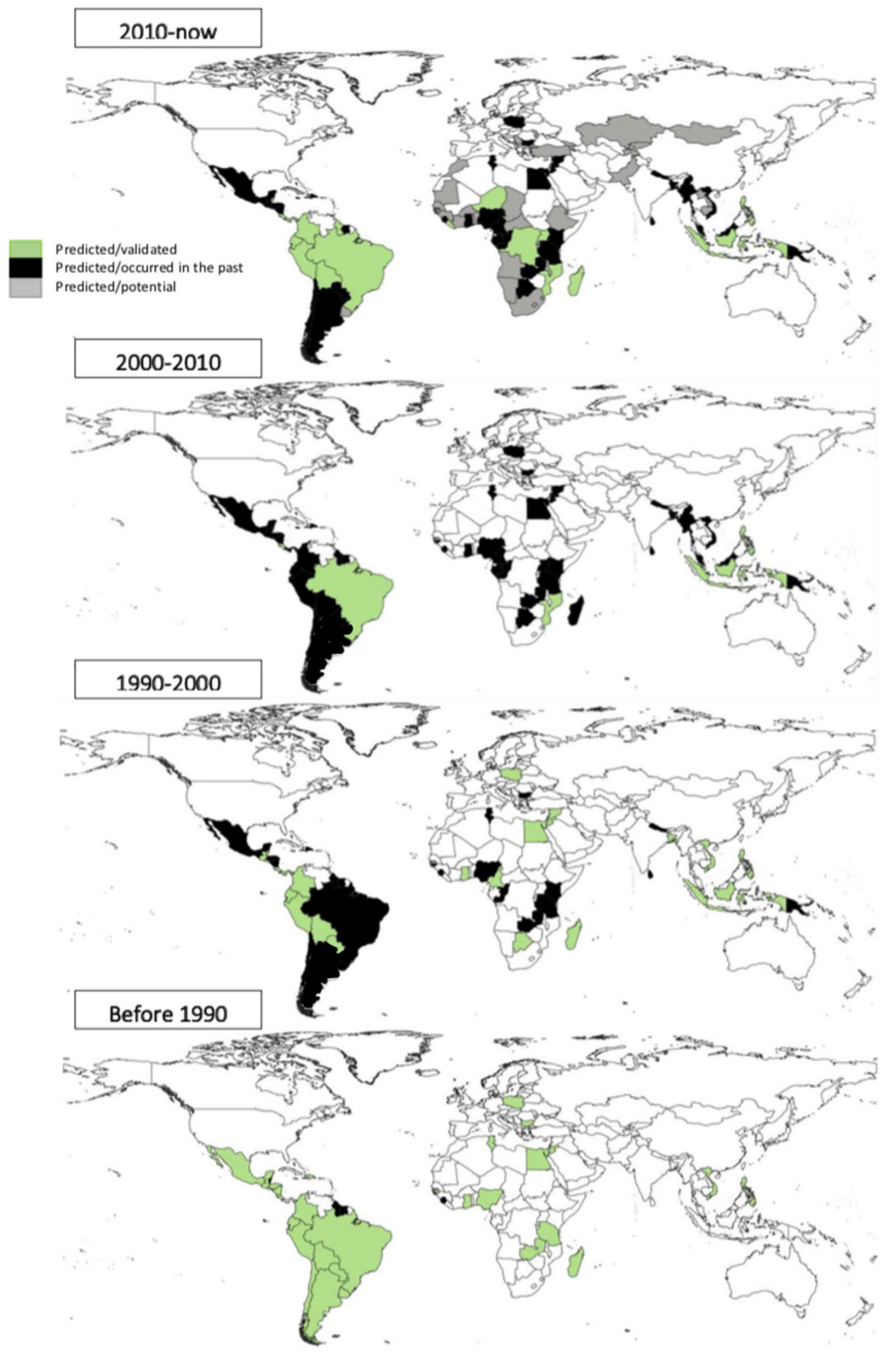
Temporal breakdown of DNS uptake in 257 countries (according to prompt-tuned GPT-4). The timeline is divided into four sub-periods: early uptakes (before the 1990s), 1990–2000, 2000–2010, and 2010 to the present day.

To facilitate the interpretation of results returned by our generative algorithm, we also broke down the entire period of this debt instrument into four sub-periods: before the 1990s (early uptakes), between 1990 and 2000, 2000–2010, and the most recent span between 2010 and today (Figure 5). The systematic analysis of 195 nations over the past decade has delineated 21 countries as optimally suited for DNS initiatives. These countries (Costa Rica, Brazil, Philippines, Madagascar, Ecuador, Colombia, Bolivia, Peru, Belize, Solomon Islands, Guyana, Panama, Niger, Liberia, Mozambique, Uganda, Seychelles, Indonesia, Vanuatu, Democratic Republic of the Congo, and Sao Tome and Principe) are characterized by their rich biodiversity and established engagement with conservation-oriented financial mechanisms. Notably, 57.1% of these nations possess a continuous history of debt-related subsidies dating back to the 1980s, indicating a sustained commitment to environmental conservation funding.

Conversely, 58 nations have been categorized as conditionally suitable by our methodology, where only two, Uruguay and Guinea-Bissau, have demonstrated a comparable legacy of conservation funding. While these countries may exhibit potential for DNS, their track record in conservation finance is not as firmly established, or they may face other deterring factors such as political instability or limited institutional capacity.

The exclusion of 35 countries from the list of suitable candidates for DNS, despite their previous engagement prior to 2010, can be attributed to a complex interplay of economic, political, and environmental factors. A pertinent example is Argentina, which, as of 2023, is embroiled in economic turmoil characterized by soaring inflation and onerous sovereign debt, diminishing its capacity to prioritize conservation finance mechanisms such as DNS. Chile's situation contrasts with Argentina's, as it boasts a relatively stable economy and a lower debt-to-GDP ratio within the Latin America region, potentially reducing the necessity or attractiveness of DNS as a fiscal tool. Honduras is a further example, as it is confronting issues of political instability and governance that could impede the consistent application of environmental priorities, thus affecting its suitability for DNS. For Poland, an EU member state with growing economic stability and access to alternative European conservation funds, the need for DNS may have diminished compared to the era preceding 2010. Jamaica's situation could mirror this, potentially achieving a degree of debt restructuring that renders DNS less pertinent or advantageous.

These examples underscore the dynamic nature of factors influencing the aptness for DNS. Fluctuations in economic conditions, progress in debt restructuring, political will, and commitments to environmental policy are all critical determinants that can evolve and, consequently, influence a country's candidacy for these financial mechanisms over time.

Analysis of the global landscape for DNS reveals a nuanced picture of continuity and change. While 21 countries are identified as prime candidates, reflecting a commitment to leveraging fiscal instruments for conservation, there is a marked discontinuity for 35 countries previously engaged in DNS. Economic volatility, like Argentina's soaring debt and inflation, and access to alternative funds, as seen in Poland and Chile, challenge the continuity of DNS as a viable financial tool. This underscores the fragility of finance in fostering robust and resilient nature restoration, as economic and political shifts can swiftly alter nations' capacities or willingness to engage in DNS, revealing the instrument's vulnerability to broader socio-economic dynamics.

Utilizing RAG-augmented GPT-4 to summarize global information about DNS, despite integrating extensive policy documents covering biodiversity potential and socio-economic and political factors, has a number of potential limitations. The model's reliance on the widely available knowledge databases may not fully capture the latest policy changes or country-specific nuances. Furthermore, its synthesis may exhibit a tendency to oversimplify some of the complex socio-economic and environmental interdependencies, which are usually highly location-specific. Additionally, the inherent bias in the source documents and the algorithm's interpretative limitations could affect the accuracy and depth of the analysis, particularly in understanding the dynamic and evolving nature of global environmental policies and economic conditions.

## 4 Conclusions and discussion

The present study illuminates the intricate interplay between financial mechanisms and conservation efforts on a global scale, encapsulated by the application of DNS. Our analysis, using generative AI on publicly mined datasets, indicates a discernible pattern wherein a subset of countries continues to exhibit suitability for DNS, suggesting a persistent recognition of the value of intertwining debt management with ecological conservation. However, the cessation of DNS engagement by a significant number of nations since 2010 highlights the financial instrument's vulnerability to the vicissitudes of economic stability and political commitment.

This discontinuity exemplifies the broader fragility of finance-dependent conservation strategies, where the robustness and resilience of nature restoration efforts are susceptible to the flux of geopolitical and macroeconomic climates. Given this context, we suggest that future research efforts should pivot toward understanding the conditions under which DNS can serve as a durable financial tool amid fluctuating economic and political landscapes. Investigative focus could also benefit from exploring alternative conservation financing mechanisms that are less contingent on the stability of national economies and more resilient to global financial uncertainties.

In terms of using RAG-augmented GPT-4 for summarizing global DNS, while comprehensive in integrating diverse policy documents, this approach exhibits certain limitations in capturing evolving nuances and policy shifts; It might be oversimplifying complex socio-economic, political and environmental interactions. Inherent biases in source material and the model's interpretation constraints could also affect the synthesis's depth and accuracy. The follow up analyses should focus on integrating real-time data and more nuanced, country-specific information to enhance understanding. Additionally, developing multi-modal algorithms with improved contextual and temporal awareness could provide a more location-specific and accurate representation of global environmental and economic policies.

## Data availability statement

The original contributions presented in the study are included in the article, further inquiries can be directed to the corresponding author.

## Author contributions

NT conceived the idea, ran experiments, and wrote up the manuscript. SF, R-RG, and CN helped revise the manuscript for re-submission. All authors contributed to the article and approved the submitted version.
